# Unlocking preservation bias in the amber insect fossil record through experimental decay

**DOI:** 10.1371/journal.pone.0195482

**Published:** 2018-04-05

**Authors:** Victoria E. McCoy, Carmen Soriano, Mirko Pegoraro, Ting Luo, Arnoud Boom, Betsy Foxman, Sarah E. Gabbott

**Affiliations:** 1 School of Geography, Geology and Environment, University of Leicester, Leicester, United Kingdom; 2 Steinmann-Institut für Geologie, Mineralogie und Paläontologie, Universität Bonn, Bonn, Germany; 3 X-ray Science Division, Advanced Photon Source, Argonne National Laboratory, Argonne, Illinois, United States of America; 4 Department of Genetics and Genome Biology, University of Leicester, Leicester, United Kingdom; 5 School of Natural Sciences and Psychology, John Moores University, Liverpool, United Kingdom; 6 Department of Epidemiology, School of Public Health, University of Michigan, Ann Arbor, Michigan, United States of America; Seoul National University College of Medicine, REPUBLIC OF KOREA

## Abstract

Fossils entombed in amber are a unique resource for reconstructing forest ecosystems, and resolving relationships of modern taxa. Such fossils are famous for their perfect, life-like appearance. However, preservation quality is vast with many sites showing only cuticular preservation, or no fossils. The taphonomic processes that control this range are largely unknown; as such, we know little about potential bias in this important record. Here we employ actualistic experiments, using, fruit flies and modern tree resin to determine whether resin type, gut microbiota, and dehydration prior to entombment affects decay. We used solid phase microextraction gas chromatography-mass spectrometry (SPME GC-MS) to confirm distinct tree resin chemistry; gut microbiota of flies was modified using antibiotics and categorized though sequencing. Decay was assessed using phase contrast synchrotron tomography. Resin type demonstrates a significant control on decay rate. The composition of the gut microbiota was also influential, with minor changes in composition affecting decay rate. Dehydration prior to entombment, contrary to expectations, enhanced decay. Our analyses show that there is potential significant bias in the amber fossil record, especially between sites with different resin types where ecological completeness and preservational fidelity are likely affected.

## Introduction

Fossil assemblages in amber provide a unique and exceptionally-well preserved record of small, soft-bodied organisms that are not typically preserved through other mechanisms of fossilization [1]. The importance of this fossil reserve is best illustrated in what it has revealed about the evolutionary history of insects: for example, it provides evidence for macroevolutionary patterns such as a mid-Cretaceous transition between two major insect evolutionary faunas, which corresponds with the gymnosperm-angiosperm shift [1]. Furthermore, preservation fidelity in amber is such that insects retain micron-scale morphological details allowing for accurate comparative anatomical studies with extant taxa: many fossil insects preserved in amber can be confidently placed into modern taxonomic groups on this basis.

However, the fossil record in amber is subject to bias, which may limit and distort the extent to which the fossil assemblage represents the living assemblage. There are two components to this bias: entrapment bias, which involves the non-random patterns by which organisms become engulfed in resin; and preservation bias, effectively the non-random patterns by which entrapped organisms decay or fossilize. To date research effort has focused on entrapment into the resin [1–8], and a number of variables such as resin viscosity, insect behaviour and habitat and plant defences have been found to be influential [1, 2]. For example, fossil assemblages in amber favour the preservation of small, terrestrial inclusions, because these are most easily entrapped in resin flows [1, 2, 4]. Subsequent to entrapment and entombment in resin it has been suggested that essentially all organisms are preserved [2]. However, recent advances in 3D scanning techniques have revealed a cryptic and surprising variability of preservation quality of internal anatomy, even where external preservation quality is close-to “life-like”. The variation ranges from internally complete fossils, retaining minute details of the most decay-prone tissues, through to fossils preserving only the most decay resistant features, to some which are hollow moulds. This indicates that decay and preservation biases are operating once carcasses are entombed in resin [1, 2, 9, 10]. Such bias may responsible for some of the patterns in the amber fossil record that cannot be explained by entrapment; for example, the occurrence of non-fossiliferous amber sites [1, 2], and taxa whose overrepresentation in amber (for example ants in some Cenozoic amber) cannot easily be explained by their behaviour [1]. The assessment of the ecological completeness of amber fossil assemblages requires understanding of bias in the preservation process of insects, as well as entrapment bias.

Here we use actualistic taphonomic experiments to determine whether decay and preservation potential of insects in amber, and therefore bias in the amber fossil record, is affected by *i*. resin-type; *ii*. dehydration prior to entombment; and, *iii*. the composition of the gut microbiota.

### Variables to account for insect preservation fidelity

Both resin type and the degree of dehydration of partially-entrapped insects prior to their full entombment have been suggested to affect preservation quality in amber [9, 11], which is why we test these variables here. We add the gut microbiome as an addition variable in our experiments for two reasons: first, in sediment-hosted exceptionally-preserved fossils it has been demonstrated to be an important control in decay and/or preservation [12–18]; and second, wild populations of fruit flies demonstrate significant variation in gut microbiota between populations [19].

1. Resin type: Resin—including modern resin, subfossil copal, and fossil amber—exhibits extensive chemical and physical variation [1, 20–23] and the preservation of fossils in amber—both presence/absence of complete taxa and their preservational quality—varies between the major amber chemical groups [9]. Thus, resin properties (physical and chemical) are likely to exert a significant control on fossil preservation in amber. Physically, resin acts as a barrier to infiltration by external decay agents such as scavengers, fungi, and bacteria [1, 2]; variations in physical properties such as permeability and viscosity may influence the effectiveness of the physical barrier. But it is the chemistry of the resin which is thought to be critical for exceptional preservation of fossils in amber [1, 24, 25], influencing decay by acting as an antiseptic, inhibiting bacterial and fungal growth and dehydrating the tissues [25–27]. The major amber chemical groups show variation in preservation quality [9]; there is generally better preservation in Class D amber (e.g. Dominican amber, with 93% of specimens preserving internal soft tissues) than in Class A amber (e.g. French Charentes amber with 0% of specimens preserving internal soft tissues [9]). However, this broad pattern is complicated by preservational variation within the major amber chemical groups, (e.g. Lebanese amber, which is in Class A, has exceptional preservation reminiscent of a typical Class D amber [9]). To date the influence of resin chemistry on decay has not been experimentally investigated. The only experiment designed to test the role of the entombing medium in preservation encased flies in wax and maple syrup, and compared them to a fly left in air [24]. Wax and maple syrup are chemically very distinct, but are not good proxies for tree resin in a number of ways including, significantly their chemistry. However, this experiment did show enhanced decay in wax and decreased decay in maple syrup compared to the control [24], suggesting that the chemical composition of the entombment media is more important than the physical barrier provided through entombment.
2. Dehydration prior to entombment: It has been suggested that dehydration of the carcass prior to entombment will result in better preservation of the resulting fossil inclusion, and may particularly promote the preservation of labile internal tissues [11]. This scenario may occur if, for example, an insect is stuck on the surface of a resin flow, dies and subsequently dehydrates in air prior to another flow entombing it.
3. Gut microbiota: Microbes are central to exceptional preservation of soft-bodied animals in sediments; their action on organically-composed anatomy may result in its loss through decay, or its preservation by mediation of authigenic mineral replacement [28, 29]. Microbes may be exogenous or endogenous, and the latter are likely to be more important in amber preservation because once entombed an organism is protected from exogenous bacteria[1, 24]. We focus here on the gut microbiota as they are known to be abundant and important in preservation [18].

## Materials and methods

### General experimental design

We designed experiments to examine whether resin type, dehydration prior to entombment and gut microbiota, influenced decay. To mimic as closely as possible natural entombment of insects in resin flies (*Drosophila melanogaster*) were embedded in freshly exuded resin, while alive but unconscious; all flies entombed in resin were stored at room temperature (~23°C) for the duration of the experiments. *Wollemia nobilis* trees were purchased from wollemipine.co.uk and *Pinus sylvestris* trees were purchased from Cole’s Plant Centre in Leicester, UK; fresh resin was collected from cuts on the trunk of each tree. The volume of resin for each experiment was not measured but was approximately equal. Flies were rendered unconscious through brief exposure to freezing temperatures, but importantly fly tissues were not frozen, as evinced by their revival after exposure if they were not quickly entombed. In all treatment groups, the fly is considered to be entrapped when it is stuck to the surface of resin, and entombed when it is entirely covered in resin [1].

### Resin type experiments

Two species of resin-producing trees, with distinct resin chemistries, were used: *Wollemia nobilis* and *Pinus sylvestris*. *Wollemia nobilis* is an Araucariacean, a family of trees whose resin (belonging to Class 1b in the mass spectroscopy resin chemistry classification scheme) is generally characterized by the presence of labanoid terpenoids and the absence of succinic acid [1, 20, 21]. There is evidence of Araucariacean resin production from the Cretaceous to the Recent [20]; a number of well-known fossiliferous amber assemblages are thought to have been produced by Araucariaceans, including Burmese amber, Lebanese amber, Spanish amber and French Charentes amber [30–37].

*Pinus sylvestris* is a Pinaceaen, a family of trees whose resin (belonging to Class 5 in a mass spectroscopy resin chemistry classification scheme) is characterized by diterpenoids such as abietane and pimarane [1, 22]. Class 5 resin in general, and *Pinus* resin in particular, does not polymerize well and is therefore very uncommon in the fossil record [1, 9, 21].

Based on taxonomy, we expected that the two resin chemistry variable states in this experiment were significantly different; we confirmed this through solid phase microextraction gas chromatography mass spectrometry (SPME GC-MS) to characterise the volatile and semi-volatile components in the resin following previous methods [38–41]. A 0.5 g sample of resin from each tree species was dried and powdered. Prior to analyses each vial of resin powder was equilibrated at 80°C for one hour and exposed to a 65 μm Polydimethylsiloxane/Divinylbenzene (PDMS/DVB) SPME fibre for one hour, and analysed on a Thermo Scientific Trace 1310 GC. A liquid injection of a standard mixture containing a series of *n*-alkanes was used to calibrate retention indices to aid in identifying the peaks. The chromatograms for each sample were imported into the program AMDIS and the major peaks in each chromatogram were identified through a National Institute of Standards and Technology (NIST) MS database search.

The chemical characterization of the *P*. *sylvestris* and *W*. *nobilis* resins confirms that they are distinct (S1 Fig, S1 Table). Of the 52 identified compounds, 18 were only found in one of the two resins (S1 Table, bolded compounds). Of the remaining 34 compounds, eight were found in much higher concentrations in one resin than the other (the difference in the total percent of that compound in the two resins > 5%, S1 Table). In total we found twenty-three compounds that differ between *P*. *sylvestris* and *W*. *nobilis* resin (S2 Table).

### Gut microbiota and dehydration experiments

The variables dehydration prior to entombment and gut microbiota and were tested on flies in *W*. *nobilis* resin. This resin was selected because it is a better proxy for amber in the fossil record and because decay in pine resin was so rapid that capture of data on decay would have been difficult using available synchrotron intervals.

In the gut microbiota treatment group we used untreated wild-type flies and flies treated with an antibiotic to alter their bacterial composition and abundances. Wild-type *Drosophila melanogaster* were collected in October 2015 by netting after the harvest in a vineyard in Market Harborough (UK). Immediately, fertilized females were isolated to generate isofemale lines. The isofemale lines were continuously maintained at 18°C in a 12hr:12hr Light:Dark (LD) cycle on standard corn meal food until the time of the experiment. Groups of 10 males (3–4 days old) were collected from 28 isofemale lines and housed in 20 antibiotic treated or 20 untreated vials (for the antibiotic treatment groups and wild type treatment groups, respectively). A solution of Ampicilin and Cloranphenicol (7mg/ml each in 50% Ethanol) was dropped (100ul) on the surface of the food in the antibiotic treated vials. Treated vials were also exposed to UV O/N. The vials were replaced every 2 days for a total of 10 days in LD12:12 at 25°C. The flies were then collected 3 hours after light on (Zeitgeber Time ZT 3) in UV treated 15ml falcon tube in ice, and were then entombed in resin.

We confirmed that the antibiotic treatment altered the wild-type fruit fly gut microbiotas, and investigated the gut microbiota composition in more detail, through 16S rRNA ion torrent sequencing of the V1 region of the gut microbiotas of ten untreated flies and nine antibiotic treated flies from the same isofemale lines as the flies used in the experiments. Operational taxonomic units were binned by 97% similarity and classified using the GreenGenes 13_8 reference database. The gut microbiota composition of the wild-type and antibiotic-treated flies were compared using nonmetric multidimensional scaling on three metrics [42, 43]: weighted UniFrac distance; unweighted UniFrac distance; and the Jaccard index. Diversity distributions (based on the Shannon index [44] for alpha diversity and the Jaccard index [43] for beta diversity) were assessed and statistically compared using Kruskal-Wallis tests [45]. All bioinformatics sequence processing up to OTU table generation was conducted using QIIME version 1.9.1 [46]. Statistical analyses and graphical output were performed with Rstudio version 0.99.484. The primary R package used for analysis of the OTU table was Phyloseq 1.12.2 [47].

Unweighted NMDS cluster analysis with either UniFrac or Jaccard distance measurements completely separates the two treatment groups (S3 Fig). In particular, there is a significant difference in alpha diversity (S5 Fig), based on the Shannon index (p = 0.04). There are similarities in microbiota community distribution of genera and phyla (S2 Fig) between the wild-type and antibiotically-treated groups. All flies, both wild-type and antibiotic-treated, had microbiotas dominated by the genus *Wolbachia* (with a relative abundance of 96% and 98%, respectively, S2 Fig, S3 Table), an endoparasitic bacterium common in the cell bodies of fruit flies and other insects [48]. *Wolbachia* is not part of the gut microbiota, but is an internal bacterium that may contribute to microbial decay. The next most common genera in both groups are *Lactobacillus*, *Acetobacter*, and *Streptococcus* (S2 Fig, S3 Table), all of which are commonly reported as dominant genera in fruit fly gut microbiotas [19].

To summarize, 16S rRNA sequencing of both types of fly showed differences in the minor components of the gut microbiome. Differences in these less-abundant genera are sufficient to separate the untreated and antibiotic treated flies into two distinct gut biota groups, which are cohesive and significantly different from each other. The antibiotic treated flies have a less diverse gut biota, containing 25 genera compared to the 44 genera in the wild-type flies (S3–S5 Figs, S3 Table).

**Fig 1:**
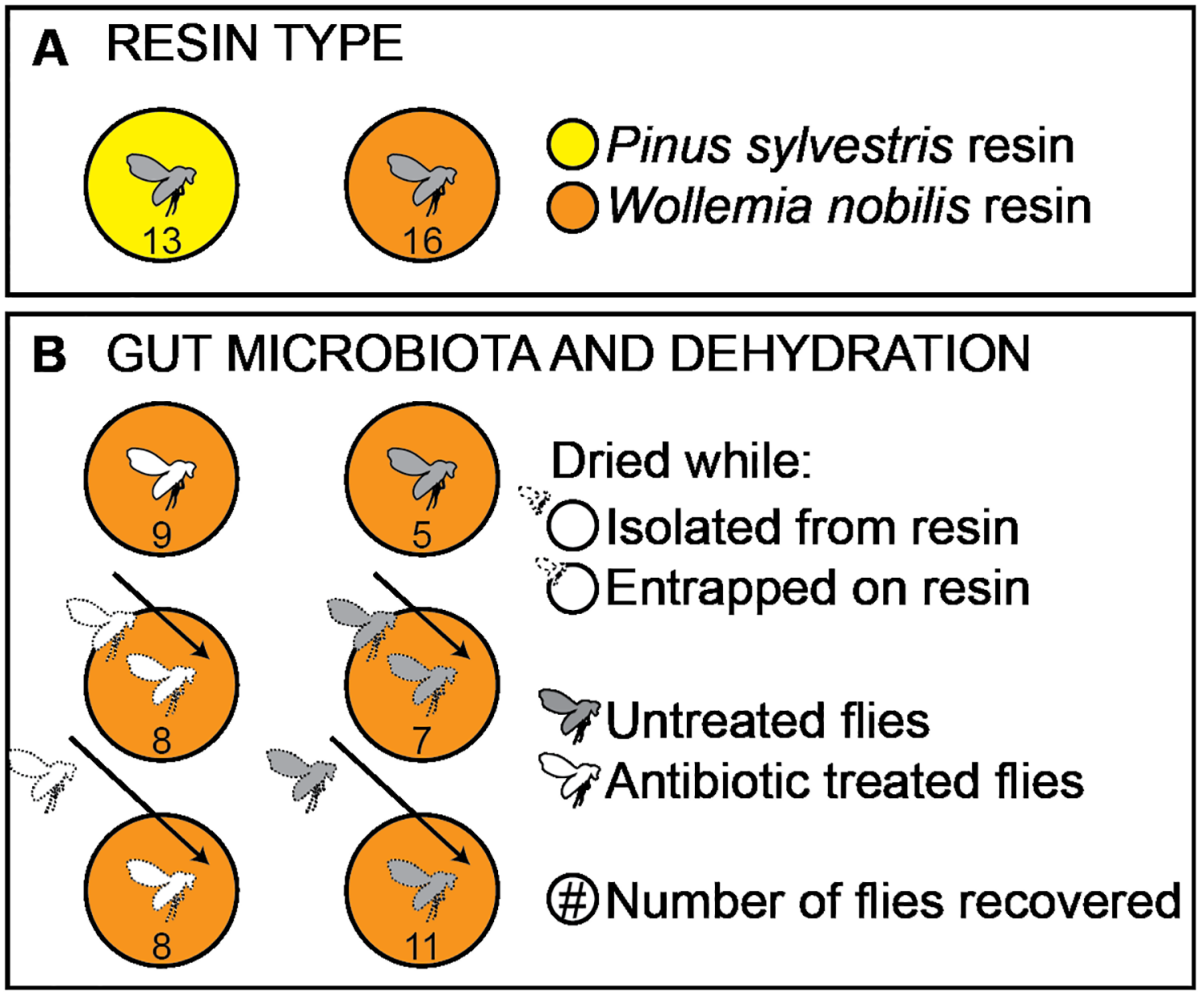
Experimental schematic. Schematic representing the variables tested (resin type, gut microbiota and dehydration state) and treatment groups in experiments. The number in each resin circle represents the number of replicates.

The effect of dehydration on decay was investigated using both untreated wild-type flies and antibiotic treated flies (Fig 1). Three states of dehydration were tested: not dehydrated; dehydrated at 50°C for three days while entrapped on the resin surface, but before entombment; and dehydrated isolated from the resin at 50°C for three days before entombment.

### Assessment of decay

The assessment of decay in different treatment groups required observation of the external and internal features of the flies in the resin, for which we used phase contrast synchrotron tomography, following standard methods for fossil inclusions in amber [10]. We imaged the experiments using different tree resins at 18 months post-entombment. At this point, the flies in *P*. *sylvestris* resin lacked all internal anatomy, so subsequent experiments to test gut microbiota and dehydration (flies in *W*. *nobilis* resin) were analysed after 2 weeks to capture possible variations in early-acting decay. Both sets of experiments (resin type and gut biota/dehydration) included untreated flies in *W*. *nobilis* resin; these directly comparable treatment groups showed very similar amounts of decay at both 2 weeks and 18 months. This suggests, that after a short burst of early decay, decay proceeds extremely slowly. Importantly, for the assessment of decay sufficient morphological loss and modification to distinguish between treatments groups occurs within a 2 week timeframe.

The synchrotron scans were analysed in the program VGStudios Max. For each fly, we noted the presence or absence of cuticle, internal tissues and ruptured abdomens. These data provide a direct indication of morphological decay. The presence of internal bubbles was also noted and provides a proxy for decay. Where bubbles occur the fly cuticle shows no significant expansion, but because bubbles take up space within the body margin internal tissues must be lost, severely diminished and/or distorted by being pushed aside by bubble formation. It was difficult from the synchrotron images to distinguish between loss or distortion of tissues, but either way preservation fidelity of internal tissues is reduced through bubble formation.

The preservation state of fruit flies in resin in each treatment group is based upon the percentage of flies showing features consistent with decay such as: loss of cuticle, loss of internal tissues, presence of bubbles within body margin, and presence of ruptured abdomens. We use Fisher Exact tests to determine if the different treatment groups are significantly associated with differences in decay. Analyses were carried out using the online calculator available at http://www.physics.csbsju.edu/stats/exact_NROW_NCOLUMN_form.html [49].

## Results

### Resin type

**Fig 2:**
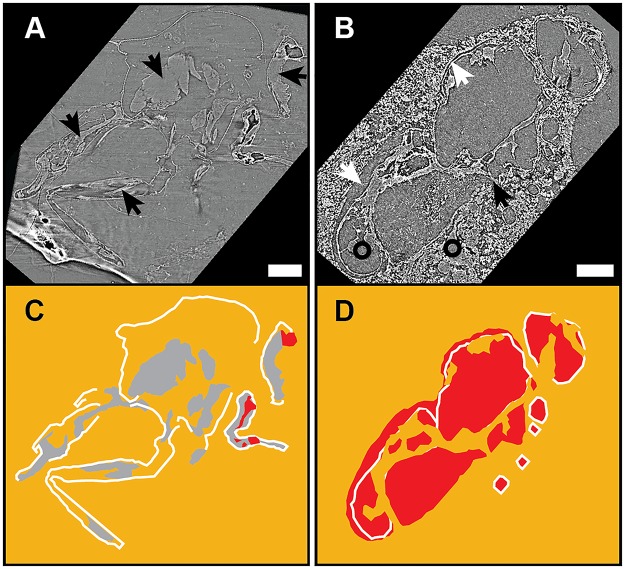
Synchrotron scans for resin chemistry experiments. Synchrotron tomographic images (A,B) and drawings (C,D) of flies in resin showing state of decay after 18 months entombment in (A) *W*. *nobilis* resin, and in (B) *P*. *sylvestris* resin. (A,C) The fly retains most of the cuticle, some of the internal soft tissues (black arrows) and bubbles within the body margin are small. (B,D) The fly retains some cuticle; there is a gap between resin and cuticle (white arrows) and extensive, and large bubbles. Internal soft tissue is absent. Instead the body margins are filled with a medium of the same density as the resin (compare the areas indicated by black circles) and which connects to the resin (black arrow), indicating that this is resin, not soft tissues. In the drawings (C,D), orange represents resin, white represents cuticle, grey represents internal soft tissue, and red represents bubbles. Scale bars = 1 mm.

After 18 months all 16 flies embedded in *W*. *nobilis* resin retained extensive preservation of cuticle and internal soft tissue in the head, thorax, abdomen and legs and ruptured abdomens. Six specimens displayed internal bubbles indicative of decay (Fig 2, Table 1, S4 Table).

**Table 1:** Results of the resin type experiments. The calculated percent of ruptured abdomens uses only specimens in which the abdomen (either ruptured or not) can be clearly seen.

Treatment group	Cuticle preserved	Internal organs preserved	Bubbles	Ruptured abdomens
*Wollemia nobilis*	16 (100%)	16 (100%)	6 (38%)	14 (100%)
*Pinus sylvestris*	13 (100%)	0 (0%)	13 (100%)	12 (100%)

In contrast, over the same time interval all 13 flies embedded in *P*. *sylvestris* resin presented as empty moulds where cuticle is frequently present but internal structures are always absent (Fig 2, Table 1, S5 Table). All flies in this treatment group showed ruptured abdomens and internal bubbles and, some (n = 10) show a gap (ranging from 5 to 50 μm) between the remaining cuticle and the resin, presumably as a result either of tissues shrinking as the fly dehydrates in the resin, or resin shrinkage due to the loss of volatiles (Fig 2, S2 Table).

Retention of internal tissues and the presence of bubbles were the decay features that were most significantly different between the flies embedded in different resins (p-values = 1.5E-08 for internal tissue preservation, and 4.1E-04, for bubble presence). Flies in *P*. *sylvestris* resin were significantly more likely to have internal bubbles and to lack internal soft tissue preservation, indicating that decay proceeds more quickly in *P*. *sylvestris* resin than in *W*. *nobilis* resin. These results are consistent with the hypothesis that resin type influences decay.

### Gut microbiota and dehydration

**Fig 3:**
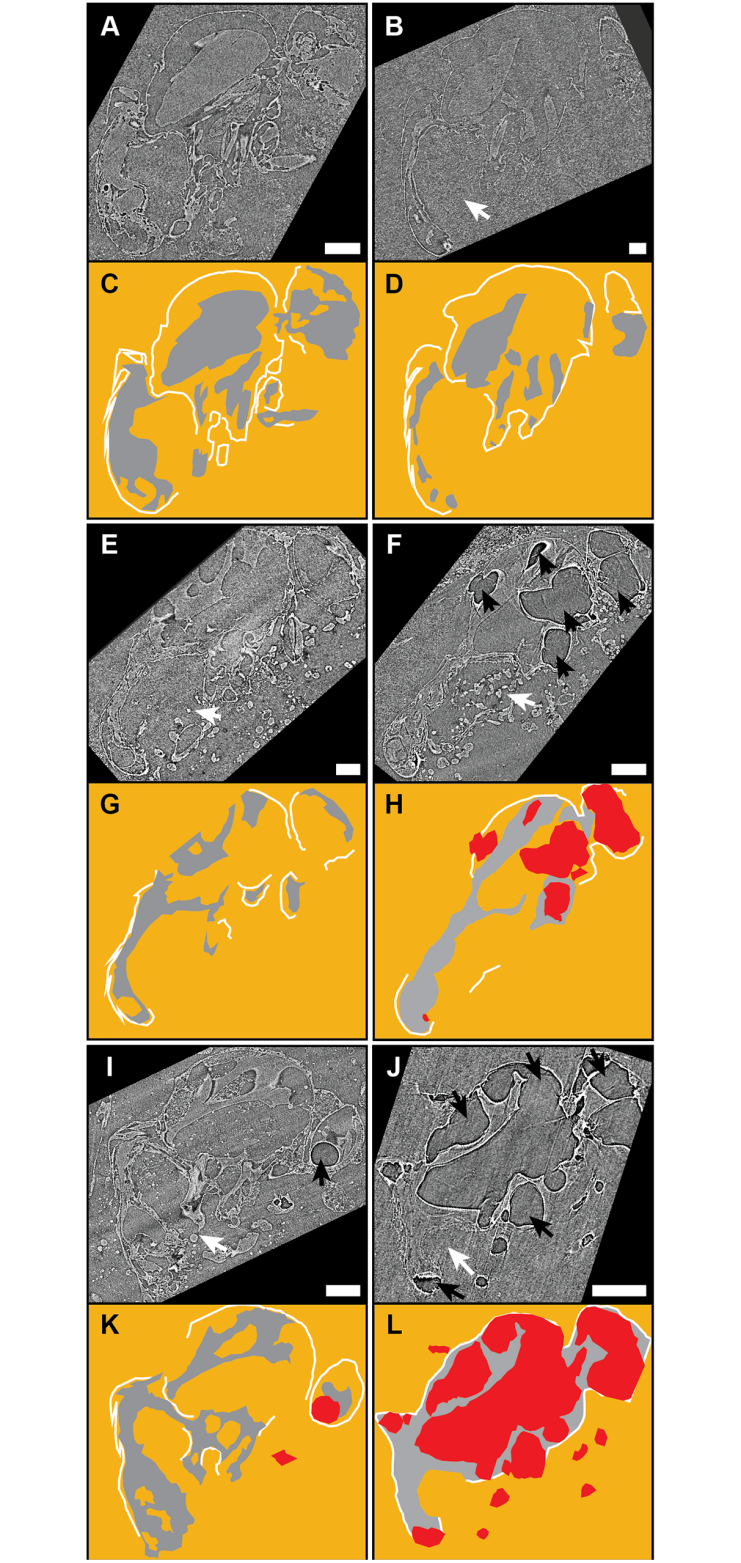
Synchrotron scans for gut microbiota and dehydration experiments. Synchrotron tomographic images (A,B,E,F,I,J) and drawings (C,D,G,H,K,L) of: (a,c) an antibiotic treated fly and (B,D) an untreated, wildtype fly, both fresh when entombed; (e,g) an antibiotic treated fly and (F,H) an untreated wildtype fly, both dried on the surface of the resin before complete entombment; and (I,K) an antibiotic treated fly, and (J,L) an untreated, wildtype fly, both dried isolated from the resin prior to complete entombment. Colours identical to Fig 2. White arrows indicate ruptured abdomens, and black arrows indicate bubbles. Scale bars = 1 mm.

After 2 weeks all flies (n = 47), regardless of treatment group, embedded in *W*. *nobilis* resin showed extensive preservation of cuticle and internal soft tissue. However, there were significant differences in the proportions of flies from each treatment group with internal bubbles and ruptured abdomens (p = 2.5E-7 and 1.9E-5, respectively), indicating variable decay between the treatment groups (Fig 3, Table 2, S3 Table).

**Table 2:** Results of the gut microbiota and dehydration experiments. The calculated percent of ruptured abdomens uses only specimens in which the abdomen (either ruptured or not) can be clearly seen.

Treatment group	Cuticle preserved	Internal organs preserved	Bubbles	Ruptured abdomens
Untreated/Not dried	5 (100%)	5 (100%)	0 (0%)	5 (100%)
Untreated/Dried on resin	7 (100%)	7 (100%)	5 (71.4%)	4 (80%)
Untreated/Dried isolated	11 (100%)	11 (100%)	11 (100%)	11 (100%)
Antibiotics/Not dried	9 (100%)	9 (100%)	1 (11.1%)	3 (37.5%)
Antibiotics/Dried on resin	8 (100%)	8 (100%)	3 (37.5%)	4 (66.7%)
Antibiotics/Dried isolated	8 (100%)	8 (100%)	8 (100%)	4 (50%)

The antibiotic treated flies had fewer bubbles and ruptured abdomens than the wild-type flies. Flies subjected to dehydration showed a greater incidence of bubbles and ruptured abdomens than flies that were not dehydrated, and this was most frequently observed in flies dehydrated in isolation prior to entombment, then in flies that were dried while entrapped on resin, and least in non-dehydrated flies (Fig 3, Table 2, S3 Table).

The most complete anatomical preservation, and lowest frequency of bubbles and ruptured abdomens was seen in antibiotic treated flies which were freshly embedded without dehydration. The most anatomical loss and highest frequency of bubbles and ruptured abdomens was seen in untreated flies that were dehydrated while isolated from the resin before entombment (Fig 3, Table 2, S3 Table).

These results are consistent with the idea that the composition of the gut microbiota influences the decay process, and therefore preservation potential. However, the idea that dehydration prior to entombment inhibits decay is not supported. Flies dried isolated from resin show more decay than those dried on the resin, where some of the fly is in contact with the resin. This is best explained because any delay in embedding a fly in resin allows decay to proceed. A corollary being that resin does have a prohibitive effect on decay.

## Discussion

### Resin type

We demonstrate experimentally that resin type operates a strong control on the decay of fruit flies: over identical time periods, flies in *W*. *nobilis* resin retained anatomical details including non-cuticular internal features, whereas flies in *P*. *sylvestris* showed poor preservation or loss of most features, including cuticle. This suggests that resin type does impart a preservational bias in the amber fossil record. This is the first, but important, step in developing a more complete understanding of the resin-type bias in the amber fossil record. To add to this we need to determine which specific characteristics of these two resins were responsible for the differences in decay. Then, different ambers could be assessed on the basis of these characteristics to determine how successfully they could inhibit the decay of labile tissues or organisms.

Resins influence decay in a number of ways: physically blocking scavengers and external microbes from the entombed carcass, dehydrating tissues to indirectly inhibit microbial activity, and/or or directly inhibiting microbial activity through antiseptic properties. The two resins used in these experiments could differ in any of these factors, for example, different permeability could vary microbe access to the carcass, and different resin chemistry could result in differential dehydration or decay-inhibition rate. There are three potential lines of evidence to understand how the volatile and semi-volatile chemical compounds that differ between the two experimental resins might influence gut microbiota activity: (1) identifying key antibiotic volatile compounds that differ between the two resins; (2) investigate the antibiotic effects of all the 23 compounds of interest on the specific bacteria present in the gut microbiotas of the fruit flies used in these experiments; and (3) comparing the overall volatile composition of the two resins to other essential oils that are more or less effective at inhibiting the activity of bacteria found in fruit fly gut microbiotas. Here we examine the literature on all three lines of evidence.

In essential oils, phenolic compounds such as thymol and carvacrol are considered to be key compounds for robust antibacterial activity [50–52]. These compounds in isolation show strong antibacterial properties, and are effective against a wide spectrum of bacteria [50]; they are also often enriched in essential oils that have strong antibacterial effects [51, 52]. Carvacrol is one of the 23 compounds that differ between *P*. *sylvestris* resin and *W*. *nobilis* resin (S1 and S2 Tables). It is present in *P*. *sylvestris* resin but not in *W*. *nobilis* resin, which seems to suggest that *P*. *sylvestris* resin is more effective at inhibiting gut bacteria activity than *W*. *nobilis* resin. However, carvacrol only makes up 0.07% of the *P*. *sylvestris* resin, whereas it is a major component of antibacterial essential oils (e.g. it makes up 81.4% of thyme essential oil [52]) and its antibacterial effects in isolation have been tested at 100% concentration. Therefore, the presence of trace amounts of carvacrol in *P*. *sylvestris* resin is unlikely to be significant for decay inhibition.

Many volatile compounds commonly found in essential oils have been assessed for antibacterial activity, but their effectiveness varies depending on the bacterium. For example, (-)-limonene (which is found in similar amounts in both resins used in this study), inhibits the growth of *Escherichia coli* and *Pseudomonas aeruginosa*, but does not inhibit the growth of *Lactobacillus plantarum* or *Enterococcus faecalis* [50] (two bacteria commonly found in fruit fly gut microbiotas [19]). This significantly reduces the amount of data we have to determine the antibacterial activity of the two resins: only six of the 23 compounds of interest—carvacrol, carvacrol methyl ether, and 3-carene, (-)-terpin-4-ol, sabinene, and α-pinene—have been studied, and their antibacterial effectiveness has only been assessed against 1 (*L*. *plantarum)* of the many bacteria (at least 44 genera) found in our fruit fly gut microbiotas (S6 Table). In our analysis, we only identified the gut microbiota bacteria to the genus level; however the genus *Lactobacillus* was found in our samples and *L*. *plantarum* is a very common species in a fruit fly gut [19]. *Pinus sylvestris* resin is enriched relative to *W*. *nobilis* resin in carvacrol, carvacrol methyl ether, and 3-carene: carvacrol and carvacrol methyl ether inhibit *L*. *plantarum*; and 3-carene does not inhibit *L*. *plantarum*. *Wollemia nobilis* resin is enriched relative to *P*. *sylvestris* resin in (-)-terpin-4-ol, sabinene, and α-pinene: (-)-terpin-4-ol inhibits *L*. *plantarum*; sabinene and α-pinene do not inhibit *L*. *plantarum*. These results only illustrate a tiny fraction of the complex chemical interactions between the resin and gut microbiota activity, and they are therefore inconclusive. Based on this we once again cannot determine which resin should have a stronger influence on decay due to the interaction of volatile compounds and gut microbiota activity.

Mixtures of volatile compounds may also inhibit bacterial activity, and so we compared the resin composition to the composition of essential oils that do or do not inhibit the activity of *L*. *plantarum*. As with investigating the compounds individually, this only reflects a small proportion of the complexity: only 10 compounds were included to describe the chemical composition of each sample; and the samples were tested against only one component of the fruit fly gut microbiota (*L*. *plantarum)*. Multiple correspondence analysis (MCA) does completely separate the essential oil samples into those that do and do not inhibit *L*. *plantarum* activity (S6 Fig). However, the two resin samples do not fall into either of these groups. Moreover, it is not clear which group the samples resemble more closely; depending on whether inhibiting activity increases along coordinate 1 or at a slight angle to coordinate 1 (S6 Fig), either *P*. *sylvestris* resin or *W*. *nobilis* resin is more closely allied with the essential oils that inhibit *L*. *plantarum* activity. So once again, the results are inconclusive as to which resin should more effectively inhibit decay. In summary, only a small subset of the resin chemical compounds, and a small subset of the fruit fly gut microbiota, have been previously investigated, and therefore the published literature does not capture the full complexity of the interactions between resin chemistry and gut microbiota activity. Moreover, even at this oversimplified level, the results of our experiments are inconclusive as to which chemical compounds and which gut bacteria have the strongest influence on decay.

Although we cannot explain how specific compounds within resins alter decay trajectory, we can illuminate some of the ways in which resin chemistry is influenced that likely contributes to resin-chemistry biases in the amber fossil record. Resin chemistry is most obviously influenced by the taxonomy of the tree and environmental factors [53, 54]. But, even small chemical differences in resin chemistry between closely related trees may be important. Such chemical distinction is thought to be strongly controlled by herbivore faunal composition, [38, 53], suggesting that even the make-up of plant-eating animals in an ecosystem may influence whether or not that fauna is preserved in amber.

That resin type strongly influences preservation potential means that the diversity of taxa recorded by amber is likely to be related to the tree/resin type at any locality; this may explain the major preservational patterns (such as which sites have fossils, and which sites have the best preserved fossils) in the amber fossil record, in both the presence/absence of fossils or the presence/absence of labile internal tissues [1, 2, 9]).

### Gut microbiota

Our experimental results indicate that very small variations in the gut microbiota influence decay. This is important because gut microbiota in fruit flies are easily altered, allowing for the potential to create a systematic biases in the amber fossil record. Wild populations of fruit flies demonstrate significant variation in gut microbiome between populations, which may be based on the food available in the region [19]; this could result in different populations of the same species having different fossilization potential when entombed in amber. In addition, because the gut microbiota must be constantly replenished through eating [19] an organism that has not fed for recently may have no gut biota. In fact, in laboratory analyses of gut microbiota, simply switching flies to a new food medium is enough to significantly reduce the density of gut bacteria, even without a period of starvation [55]. Therefore, even within the same species the types, consistency and quantities of food available in a certain environment, or across different environments may influence its preservation potential in amber. A gut microbiota bias could explain preservational variation between amber sites that are geographically localized and chemically similar, but which vary in presence or absence of an entombed fauna. For example, only 17 of over 300 Lebanese amber outcrops contain fossils [35]; and only seven out of more than 100 Spanish amber outcrops contain fossils [56]. The similarities in amber chemistry suggest that resin chemistry was not sufficiently different between these sites to explain preservation variation. It is possible that gut microbiotas may have varied between these populations, and this might explain the presence or absence of fossils.

### Conclusions

To conclude, the surprising variability in preservation quality of insects in amber means that to read this fossil treasure trove correctly, using it to investigate terrestrial ecosystems and insect evolution, we must understand the biases that operate in creating such fossils. Here we show that decay experiments provide a useful platform with which to investigate bias between different aspects of the amber fossil record.

## Supporting information

S1 Fig Chromatograms and selected peaks. Chromatograms (A, C) and selected peaks highlighting the compounds of interest (B, D) from SPME GC/MS analysis of *Wollemia nobilis* resin (A, B) and *Pinus sylvestris* resin (C, D). Each selected peak is numbered using the labels from S2 Table.
(TIF)

S2 Fig Community distribution of gut microbiota in different treatment groups. (A) Genus distribution. (**b**) Phylum distribution. There is very little difference in community distribution at the genus or phylum level between the untreated and antibiotic treated flies. The biggest community differences are in the least-abundant genera.
(TIF)

S3 Fig NMDS clustering of fruit flies based on various measures of gut microbiota similarity. (A) Weighted UniFrac distance. The untreated and antibiotic treated gut microbiota communities are all dominated by one genus, *Wolbachia*, and therefore they cannot be distinguished in a weighted NMDS analysis. (B) Unweighted UniFrac distance. The two treatment groups are distinct in an unweighted NMDS analysis using the UniFrac distance, because there are meaningful differences in the less abundant components of the gut microbiotas. (C) Jaccard distance. Once again, an unweighted distance metric also distinguishes the two gut microbiota treatment groups.
(TIF)

S4 Fig Jaccard index of beta diversity. Comparing the diversity within and between the two fruit fly treatment groups, indicating that the groups are cohesive and distinct from each other. Cohesive: there is no significant difference in gut microbiota similarity within each treatment group (p = 0.066). Distinct: there is a significant difference in the similarity within each group and the similarity between the groups (p-values of 0.001 and <0.001).
(TIF)

S5 Fig Shannon alpha diversity. Showing the difference in gut microbiota diversity between the untreated flies and the antibiotic treated flies. This difference between the treatment groups is significant, with a p-value of 0.0412, indicating the two treatment groups are distinct.
(TIF)

S6 Fig MCA analysis of *P*. *sylvestris* and *W*. *nobilis* resin and various essential oils whose antibacterial properties are known. Note that the MCA does separate the essential oils into those that do and do not inhibit the activity of *L*. *plantarum*. However, the resins do not fall clearly into either group. (A) MCA, assuming that coordinate 1 represents the antibacterial effectiveness of the compound against *L*. *plantarum*, in which case *P*. *sylvestris* resin seems to be more likely to effectively inhibit decay. (B) MCA assuming that antibacterial effectiveness corresponds to a vector intermediate between coordinates 1 and 2, in which case *W*. *nobilis* resin seems likely to be slightly more effective at inhibiting decay.
(TIF)

S1 Table Chemical composition of *Pinus sylvestris* and *Wollemia nobilis* resin. Compounds in bold are only present in one of the two resins. The compounds are ordered based on the difference in % area between the two resins; the first rows are more concentrated in *P*. *sylvestris* resin, and the bottom rows are more concentrated in *W*. *nobilis* resin.
(XLSX)

S2 Table Specific compounds of interest. Chemical description of the 23 compounds that differ between *P*. *sylvestris* and *W*. *nobilis* resin.
(XLSX)

S3 Table Gut microbiota composition. Relative abundance of microbial taxa identified by treatment group and mock community, in order of abundance.
(XLSX)

S4 Table All data from resin chemistry experiments. This details the presence/absence of various features in each body part, for each fly. ‘?’ indicates that the feature cannot be resolved, typically because the window of the scan does not encompass the entire fly.
(XLSX)

S5 Table All data from gut microbiota/dehydration experiments. This details the presence/absence of various features in each body part, for each fly. ‘?’ indicates that the feature cannot be resolved, typically because the window of the scan does not encompass the entire fly.
(XLSX)

S6 Table Influence of resin chemical compounds on the components of the fruit fly gut microbiota. Only six of the 23 potentially important compounds have been studied for their antibacterial effects, and only on one species of the 44 identified bacteria genera from the fruit fly gut microbiotas. No research has been done on how these compounds would influence the fungi or the *Wolbachia*, both of which also are present in fruit fly guts; *Wolbachia* dominates the fruit fly microbiota.
(XLSX)

S7 Table Chemical composition of various essential oils compared to the chemical composition of *P*. *sylvestris* and *W*. *nobilis* resin. All essential oil samples were purified from plants using hydrodistillation, and then analysed with GC-MS.
(XLSX)

S8 Table Detailed taxonomic information and genbank accession numbers for gut microbiota sequencing. (XLSX)
