# Supplementary figures and images for: Unlocking preservation bias in the amber insect fossil record through experimental decay
Source: PLoS One. 2018 Apr 5;13(4):e0195482. doi: 10.1371/journal.pone.0195482 (PMC5886561; doi:10.1371/journal.pone.0195482)

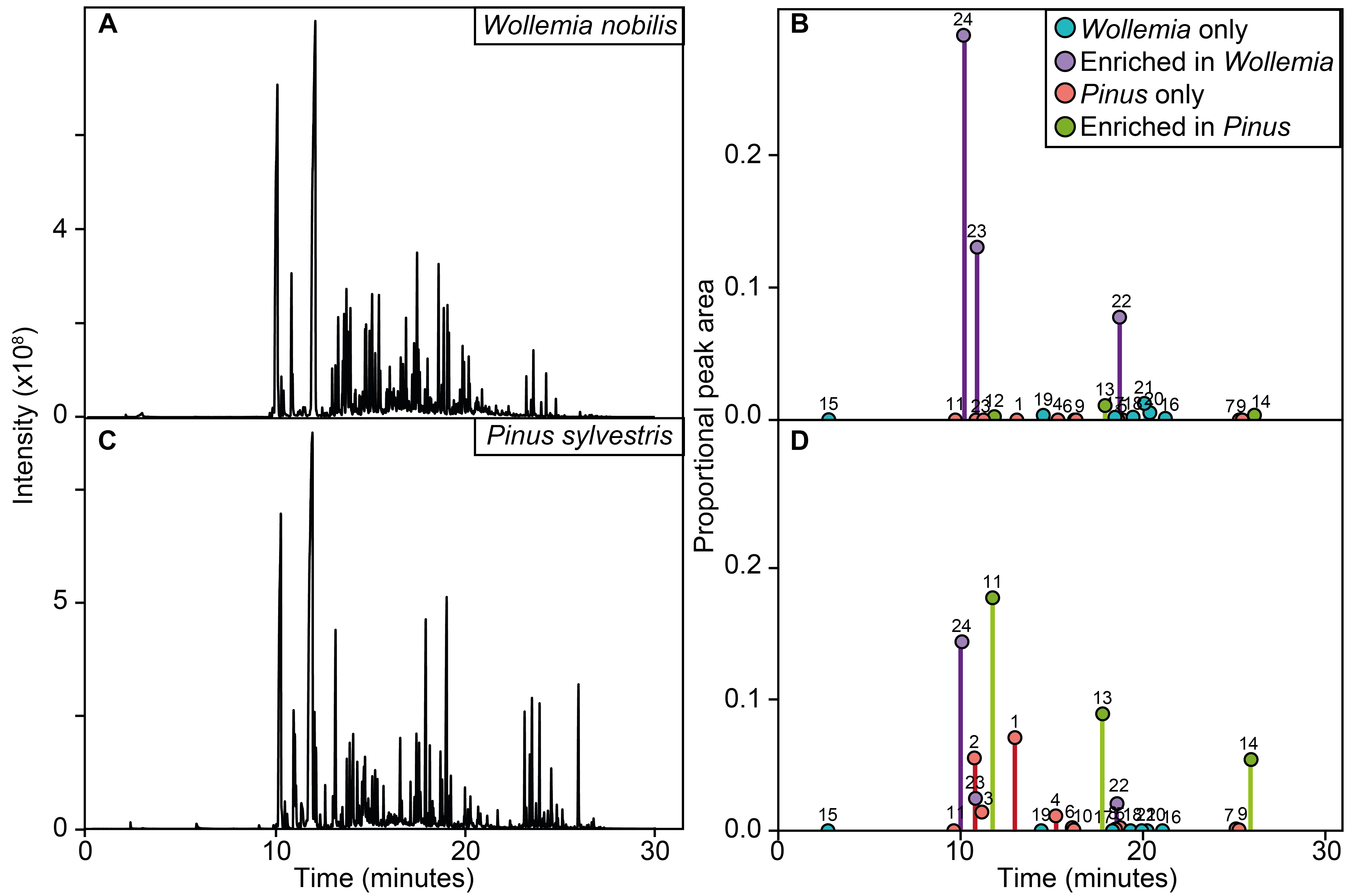

Supplement: S1 Fig — Chromatograms (A, C) and selected peaks highlighting the compounds of interest (B, D) from SPME GC/MS analysis of Wollemia nobilis resin (A, B) and Pinus sylvestris resin (C, D). Each selected peak is numbered using the labels from S2 Table. (TIF) [file pone.0195482.s001.tif]

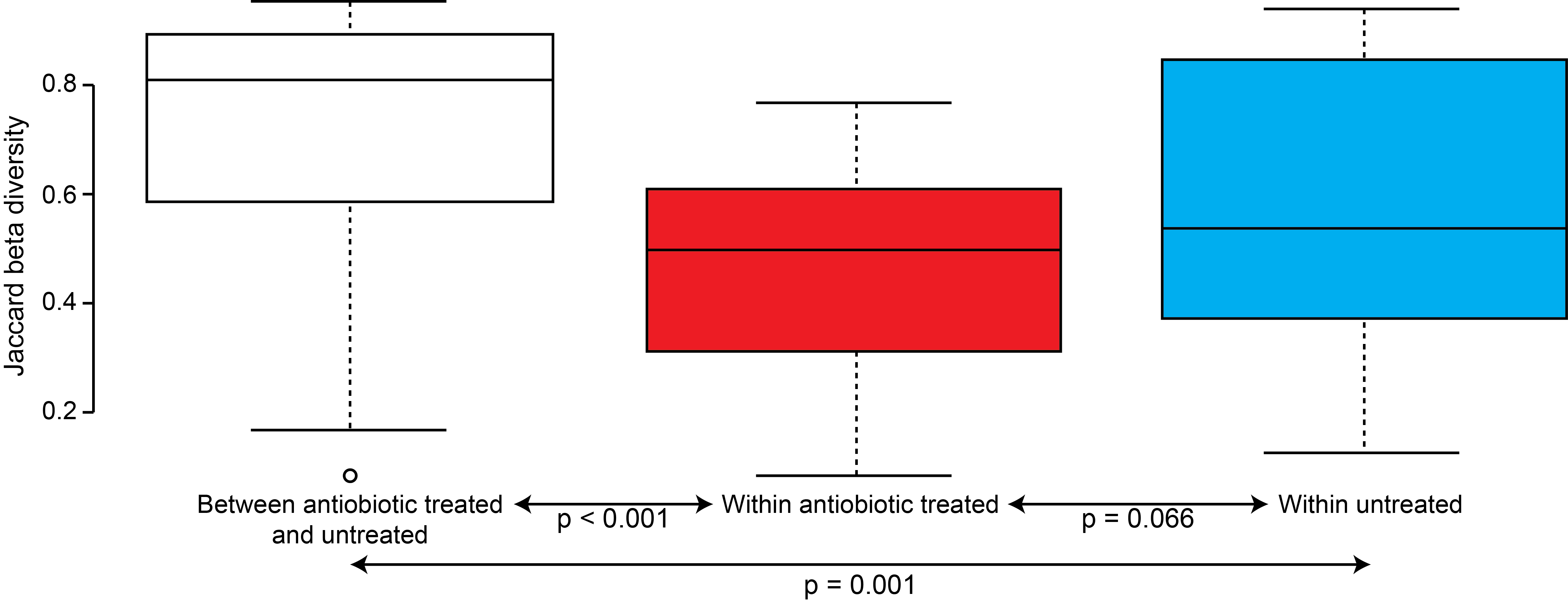

Supplement: S4 Fig — Comparing the diversity within and between the two fruit fly treatment groups, indicating that the groups are cohesive and distinct from each other. Cohesive: there is no significant difference in gut microbiota similarity within each treatment group (p = 0.066). Distinct: there is a significant difference in the similarity within each group and the similarity between the groups (p-values of 0.001 and <0.001). (TIF) [file pone.0195482.s004.tif]

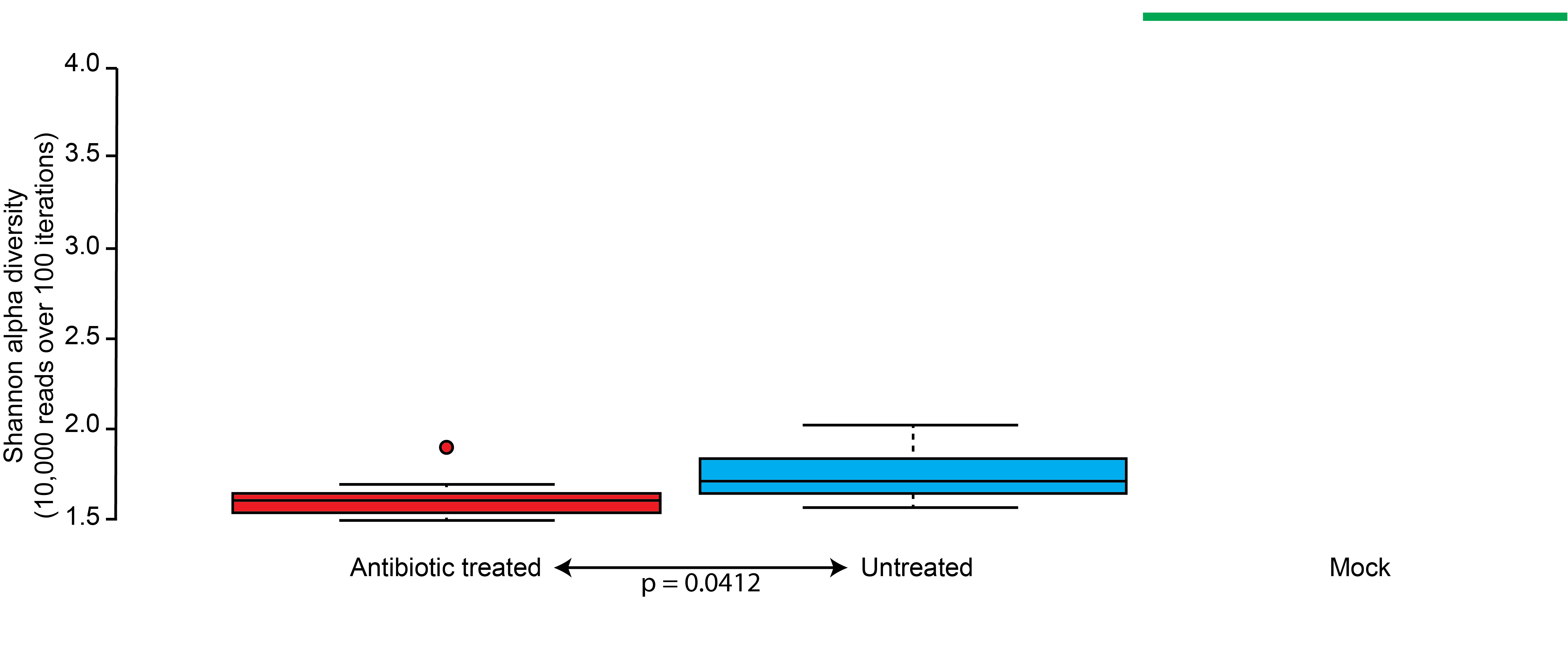

Supplement: S5 Fig — Showing the difference in gut microbiota diversity between the untreated flies and the antibiotic treated flies. This difference between the treatment groups is significant, with a p-value of 0.0412, indicating the two treatment groups are distinct. (TIF) [file pone.0195482.s005.tif]

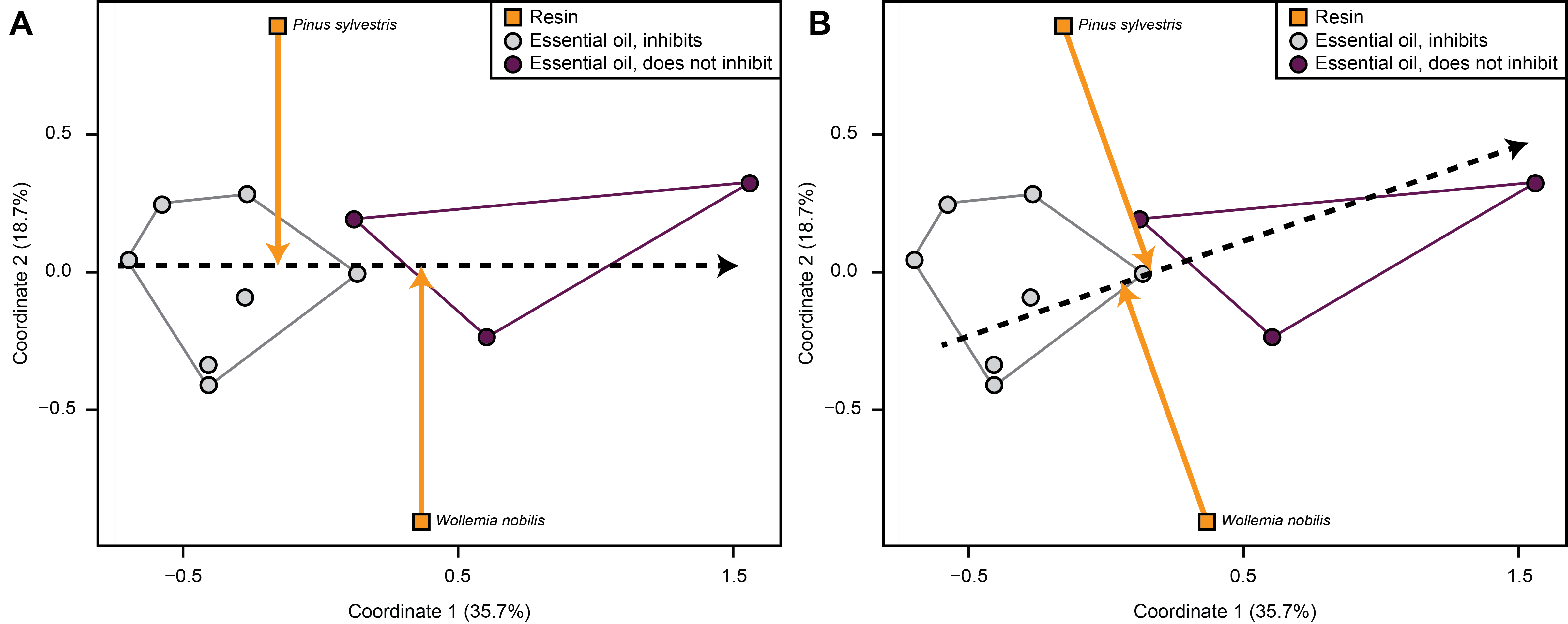

Supplement: S6 Fig — Note that the MCA does separate the essential oils into those that do and do not inhibit the activity of L. plantarum. However, the resins do not fall clearly into either group. (A) MCA, assuming that coordinate 1 represents the antibacterial effectiveness of the compound against L. plantarum, in which case P. sylvestris resin seems to be more likely to effectively inhibit decay. (B) MCA assuming that antibacterial effectiveness corresponds to a vector intermediate between coordinates 1 and 2, in which case W. nobilis resin seems likely to be slightly more effective at inhibiting decay. (TIF) [file pone.0195482.s006.tif]
